# The coalitional politics of the European Union’s environmental forest policy: Biodiversity conservation, timber legality, and climate protection

**DOI:** 10.1007/s13280-021-01644-5

**Published:** 2021-10-10

**Authors:** Metodi Sotirov, Georg Winkel, Katarina Eckerberg

**Affiliations:** 1 Faculty of Environment and Natural Resources, Institute of Environmental Social Sciences, Chair of Forest and Environmental Policy, University of Freiburg, Tennenbacher Str. 4, 79106 Freiburg, Germany; 2 Governance Programme Bonn, European Forest Institute, Platz der Vereinten Nationen 7, 53113 Bonn, Germany; 3 Department of Political Science, Umeå University, 90187 Umeå, Sweden

**Keywords:** EUTR/FLEGT, LULUCF, Multi-level governance, Natura 2000, Policy beliefs, Policy change

## Abstract

European forest policymaking is shaped by progressing European integration, yet with notable ideological divisions and diverging interests among countries. This paper focuses on the coalitional politics of key environmental forest issues: biodiversity conservation, timber legality, and climate protection policy. Combining the Advocacy Coalition Framework and the Shifting Coalition Theory, and informed by more than 186 key informant interviews and 73 policy documents spanning a 20-year timeframe, we examine the evolution of coalitional forest politics in Europe. We find that the basic line-up has remained stable: an environmental coalition supporting EU environmental forest policy integration and a forest sector coalition mostly opposing it. Still, strategic alliances across these coalitions have occurred for specific policy issues which have resulted in a gradual establishment of an EU environmental forest policy. We conclude with discussion of our findings and provide suggestions for further research.

**Supplementary Information:**

The online version contains supplementary material available at 10.1007/s13280-021-01644-5.

## Introduction

Forest issues in the European Union (EU) and its Member States (MS) have been subject to policy controversy for decades. In particular, biodiversity conservation and forest management are disputed along an ideological forestry-environmental division spanning across the EU multi-level governance system. The dispute concerns trade-offs regarding multiple forest ecosystem services, such as biomass for the bioeconomy and rural development (Söderberg and Eckerberg 2013; Winkel 2017), biodiversity (Sotirov 2017), or climate change-related services (Nabuurs et al. 2013).

A review of current research reveals that the regulation of such trade-offs is subject to a complex and still poorly understood EU multi-level policy framework: The treaties establishing the EU make no legal provision for a specific common EU forest policy and, so far, only non-binding strategies and action plans have been agreed upon (Aggestam and Pülzl 2020). Attempts for establishing an EU-wide specific forest policy have met with resistance, particularly from forest-rich MS with economically important forest sectors such as Finland, France, Germany, Poland, and Sweden (Winkel and Sotirov 2016). Consequently, the control of forest policy and legislation has seemingly remained under the sovereignty of each MS (Pülzl et al. 2018).

Yet a multitude of other established EU sectoral policies is impacting national forest policies (Pülzl et al. 2018). This includes the Common Agriculture and Rural Development Policy (CAP), Renewable Energy Policy (Renewable Energy Directive: RED I and II), the EU’s Nature Protection and Biodiversity Policy (Habitats and Birds Directives, also known as the Nature Directives), the EU’s Water Protection Policy (Water Framework and Drinking Water Directives), the EU’s Forest Law Enforcement, Governance and Trade Policy (EU FLEGT VPAs/EU Timber Regulation), and the EU’s Climate Action in the Land Use, Land Use Change and Forestry (LULUCF) Sector (EU’s LULUCF Regulation) (Hix 2005; Wolfslehner et al. 2020).

This EU forest-related policy framework has emerged despite policy controversies and blockages, including failed attempts to establish a common EU forest policy (Winkel and Sotirov 2016; Pülzl et al. 2018), a European Forest Information System (Baycheva-Merger and Sotirov 2020), and a Pan-European Legally Binding Agreement on Forests (Edwards and Kleinschmit 2013). Scholars have analyzed such controversies over decision-making competencies between the EU and national governance levels and across sectors in issue areas such as forestry and biodiversity (Eckerberg and Sandström 2013; DeKoning et al. 2014; Borrass et al. 2015; Sotirov and Storch 2018), forestry and bioenergy (Söderberg and Eckerberg 2013; Sotirov and Storch 2018), and forestry and water (Baulenas and Sotirov 2020).

But how can we explain the development of EU environmental forest policies despite such controversies and failures? Taking up a coalitional policy analysis perspective, this paper investigates this issue addressing the following research questions:

- Which policy advocacy coalitions have influenced EU environmental forest policy development?
- Which coalitional dynamics can explain EU environmental forest policy development?

## Theoretical framework

Several theoretical approaches are available for studying policy making. Among these, the Advocacy Coalition Framework (ACF, see Sabatier 1988; Sabatier and Jenkins-Smith 1999; Sabatier and Weible 2007; Jenkins-Smith et al. 2014) is deemed as one of the most elaborate theoretical lenses (Sotirov et al. 2011). While most of the more than 230 ACF applications focus on environmental and natural resource policymaking in North America and Europe, EU environmental forest policy issues have not been addressed thus far. In this paper, we use an ACF perspective and complement it with the Shifting Coalition Theory (SCT) (Sotirov and Memmler 2012; Sotirov and Winkel 2016) that has been acknowledged as an important theoretical innovation in ACF scholarship to explain coalition formation in EU forest policy making (Jenkins-Smith et al. 2014).

The ACF and the SCT share the idea that policy outcomes are the result of coalitional politics and political institutions. A further commonality is their assumptions about the pivotal role of actors’ beliefs and the strategic use of power resources that shape coalitional politics and institutional venue shopping (Sotirov and Memmler 2012; Sotirov and Winkel 2016).

The two perspectives differ and thus complement each other in their explanation of policy development. The ACF emphasizes enduring *competition* among *stable coalitions* holding opposing normative policy beliefs that result in policy *stability*. The main driving forces are social psychological mechanisms such as diverging human perceptions caused by cognitive biases. Substantial policy change is only possible when a coalition changes the power balance e.g., through skilfully exploiting events such as changes in government, socio-economic crisis, or natural disasters (Sabatier and Jenkins-Smith 1999; Sabatier and Weible 2007; Jenkins-Smith et al. 2014). The SCT highlights (temporary) *shifting coalitions* through *strategic alliance building* across coalitions relating to specific policy issues that occur either simultaneously or sequentially over time. Alliances are enabled through shared or complementary beliefs or strategic interests in a specific policy regulation (Sotirov and Memmler 2012). They can result in substantial *policy change* (Sotirov and Winkel 2016). For our subsequent analysis, this results in two hypotheses relating to the ACF propositions:

### Hypothesis 1 (H-1)

EU environmental forest policy is generally characterized by a stable line-up of competing advocacy coalitions connected to opposing policy core beliefs (“Adversarial coalitional politics” Hypothesis).

### Hypothesis 2 (H-2)

Change in EU environmental forest policy is rare unless a coalition skilfully exploits changes in the framework conditions for a policy reform (“Policy stability” Hypothesis).

Our third hypothesis draws on the SCT propositions:

### Hypothesis 3 (H-3)

Depending on the policy issue at stake, dynamic processes of (re-) building strategic alliances occur across different advocacy coalitions (“Shifting coalitions” Hypothesis).

An advocacy coalition is composed of state and non-state actors that coordinate actions to implement their shared beliefs into policy. These beliefs include, for instance, basic value priorities regarding groups or other entities whose welfare is of greatest concern, beliefs about the nature and severity of the policy problem and about who is responsible for appropriate problem solutions (Sabatier and Jenkins-Smith 1999; Weible et al. 2009).

Coalitions strategically use power resources and influence decision-making venues to advance their beliefs in policy making. Coalitions’ strategic actions rely on six types of power resources: (a.) decision-making authority, (b.) money, (c.) information, (d.) supportive public opinion, (e.) mobilization of supporters, and (f.) skilful leadership (Sabatier and Weible 2007). Both ACF and SCT share the argument that the availability of strategic power resources is shaped by the institutional framework in which coalitions operate. Accordingly, changes in the institutional framework conditions can (re-)shape the availability of power resources (Sabatier and Weible 2007; Sotirov and Winkel 2016).

The EU multi-level governance system offers plenty of venues to influence policy development (Sabatier 1998). Agenda-setting and policy formulation are usually dominated by the European Commission (“the Commission”). It is organized like a domestic government, with a core executive (the College of Commissioners) focusing on the initiation of legislation and arbitration in the legislative process, and a bureaucracy (the directorate generals, DGs) preparing laws and monitoring law enforcement (Hix 2005). In the phase of policy adoption, the Council of the European Ministers (“the Council”) acts as an assembly of member state executives in charge of a given issue area, and the European Parliament (“the Parliament”) shares decision-making competency with the Council. At this stage, the Commission plays a role as consensus-seeking broker between the Council and the Parliament that are usually negotiating with each other (Marks et al. 1996; Hix 2005).

In the phase of policy implementation, decision-making responsibilities are shared between the Commission and national governments, while involving also sub-national authorities. The European Court of Justice (“the Court”) frequently supports domestic implementation of EU policy through EU compliance and enforcement actions under the legal threat of infringement procedures. The Commission also assesses experiences with the domestic implementation of existing EU policies, evaluates their effectiveness and proposes further EU policy changes (Marks et al. 1996).

In result, EU policies are developed in the highly pluralistic EU multi-level policymaking system characterized by openness and consensus needed for policy change (Sabatier 1998). The variety of policy actors involved, the EU institutions vested with decision-making authority, and the many institutional venues usually results in political compromises (Peterson 1995). A diversity of institutional venues provides advocacy coalition with an opportunity to bypass powerful political adversaries and to place their policy ideas at new venues, thereby forming strategic cross-coalitional “winning alliances” to achieve policy change (Pralle 2003; Sotirov and Winkel 2016). This observation results in our last hypothesis:

Hypothesis 4 (H-4): Changes in EU environmental forest policy entail a mixture of core beliefs from various coalitions that are dependent on each other to influence policy in the complex EU policy making system (“Institutionalist hypothesis”).

## Materials and methods

Our policy research draws on qualitative content analysis of interviews and documents. References to those numbered interviews (I1, I2, I3, etc.) and documents (D1, D2, D3, etc.) can be found in the text, especially at the end of each paragraphs and in the process-tracing tables. Further details are placed in the Electronic Supplementary Material (ESM), Tables S1–S6. For all the three case studies, 186 semi-structured interviews were conducted with key informants representing EU institutions, MS authorities, environmental NGOs, forest and landowner enterprises, timber traders, forest sector industry and scientists. Policy documents included official documents (legislation, legal drafts), coalition members’ published material (press releases, opinion papers, media articles), and previous research (scientific papers, reports) (Cresswell 2005).

The policy analysis was carried out through process tracing. Process tracing here refers to the explanation of policy development over time, identifying the causal mechanisms that link processes and causes of policymaking (X or Y) with their effects on policy development that manifest in different policy outcomes (Z) (Beach 2016). Drawing on the propositions of the ACF and SCT, we analyze the key variables identified in the theory chapter. They include adversarial coalitional politics (X_1_) or cooperative politics through shifting coalitions (X_2_), the use of different power resources (Y_1a,1b,1c_) or institutional venues (Y_2a,2b,2c_), as well as their effects on policy development such as policy change (Z_1_) or policy stability (Z_2_) (Beach and Pedersen 2013). We compare causes and outcomes across three cases (George and Bennet 2005; Yin 2009). These cases include forest biodiversity conservation (Natura 2000), timber legality/forest sustainability (FLEGT), and forest climate protection (LULUCF).

To identify coalitions, we identified policy actors’ beliefs as expressed in written statements and interviews. In addition, actors were asked to identify other actors that they cooperate with, and actors they saw as their opponents in the policy process. Cooperation among actors was also cross-checked through documents such as co-signed letters, reports, and statements (Cresswell 2005).

## Results

### Natura 2000: EU (forest) biodiversity conservation policy

#### Policy initiation

In late 1980s, an environmental coalition of non-state and state actors from national and EU levels (Table 1) successfully initiated a change in nature protection policy. Together with conservation biology scientists and public conservation authorities, a wide range of environmental NGOs provided evidence relating to an acute biodiversity crisis, emphasizing causes such as intense land use practices and insufficient protection by national level nature protection laws. Between 1986 and 1988, high-level policymakers at the Commission’s Directorate-General Environment (DG ENV) collaborated with environmental groups and drafted a new EU directive on the conservation of natural habitats and of wild fauna and flora (Habitats Directive). Supported by public opinion, the Commission justified the new legal proposal with the necessity to better implement the 1979 EU Birds Directive on the Conservation of Wild Birds and related emerging international treaties (e.g., the Convention on Biological Diversity) (D1-5; DI7-9; I15-16, I17-20, I-61).

**Table 1:** Policy advocacy coalitions in EU Natura 2000 policy, 1988–2016, and post 2018 (I1-63)

Nature conservation coalition	Policy core beliefs	Forestry and land use coalition
Biodiversity loss, unfavorable conservation status of natural habitats, flora, and fauna	Problem definition	Threat on property rights: restrictions on management, economic burdens, taking land out of production
Biodiversity, flora, fauna, habitats	Units of concern	Owners, property, bioeconomy
Intensive human use of nature by forestry, agriculture, urban infrastructure; reluctance by landowners and land users to change practices toward nature protection	Causes of the problem	Overprotection and static conservation approach; Lacking involvement of landowners and land users; Lacking funding
Foresters and all land users must take conservation objectives serious and change current practices; financial compensations for resulting restrictions from conservation management	Problem solution	Active and flexible management is needed: sustainable forest use (sustained yield). No need to change existing (sustainable) practices
European Commission (DG ENV), European Environment Agency (EEA), Council of the EU (Environment Ministers), member state environmental authorities, environmental NGOs, green and social-democratic MEPs	Members	EU Commission (DG Agriculture and Rural Development), Council of the EU (Agricultural Ministers), member state agricultural and forestry authorities, EU associations of farmers and private forest owners (CEPF, ELO, Copa-Cogega), EU association of public forestry companies (EUSTAFOR), EU forest industry associations (Cei-Boi, EOS, CEPI), EU hunters’ associations (FACE), conservative and liberal MEPs

#### Policy adoption

The negotiations on the EU Habitats Directive between the Commission and the Council started in 1988. The EU Environmental and Agricultural Council (assemblies of MS executives in charge of nature protection) officially adopted the new EU biodiversity law in May 1992 (Sharp 1998). The EU Parliament was not involved since its respective competencies (co-decision procedure) were introduced only in 1993 by the Treaty of Maastricht. A political agreement was achieved under the leadership of state actors from the environmental coalition skilfully using the venues of the EU Council Presidencies by France (second half 1989), Luxembourg (first half 1991) and the Netherlands (second half 1991). Specifically, the Dutch, Luxembourg and UK state environmental authorities were leading their respective governments’ decisions to promote the adoption of the Directive as a strategy to upscale their already existing models of protected area networks to the EU level (Liefferink and van der Zowen 2004; Borrass et al. 2015). The newly appointed UK government under PM John Major took a supportive stance on EU environmental regulation, unlike the previous Thatcher government (Fairbrass and Jordan 2003). In parallel, the French incumbent environment minister (a former Greenpeace member) helped overcome the opposition of national agricultural and hunting interest groups, while the German government abstained in the final vote due to internal political deadlock between the supportive federal ministry of environmental protection and the opposing agricultural and forestry state authorities (Wurzel 2008). An initial policy blockade by Spain and Italy was broken after domestic environmental NGOs put moral pressure on these countries, and the Commission (DG ENV) offered EU financial support (Fernandez 2003). Showing political commitment to the 1992 Earth Summit in Rio de Janeiro, the environmental coalition also included sustainable development into the Habitats Directive to win the support by some opponents. DG ENV did, however, not assess economic costs of the Habitats Directive’s implementation and argued that few economic constraints would incur for the concerned land use sectors (D1-4).

The opposition coalition was poorly organized. Forest sector interest groups from forest-rich countries (France and Germany) did not engage as they were not yet organized at the EU level (Weber and Christophersen 2002). Most other countries (Denmark, Belgium, the Netherlands and the UK) were supportive of the new EU biodiversity policy as they had already shifted their forest policy toward environmental management. Other forest-rich countries with economic forest sector interests (Austria, Sweden, Finland, and some East European countries) were not yet part of the EU-12, unlike today in the EU-27 (I27-33, I37).

The finally adopted policy represented a substantial policy change. The Habitats Directive obliged MS to designate and manage a coherent, EU-wide network of protected areas called Natura 2000. It empowers the Commission (DG ENV) to perform sufficiency checks and request improvements in selected sites. Notably, only evidence from conservation science was to be considered to protect or restore, at favorable conservation status, natural and semi-natural habitats. The Habitats Directive also obliges MS to introduce a strict system of protection for certain plant and animal species, which also applies beyond designated protected areas (D4; Borrass et al. 2015).

#### Policy implementation

Between 1993 and 2000, key members of the environmental coalition such as the Commission (DG ENV), the European Environment Agency (EEA), the European Topic Center on Biodiversity (ETSB), MS environmental public authorities as well as environmental NGOs and scientists worked together to support policy implementation. They assessed MS proposals for Natura 2000 protected areas and demanded expansions. The members of the forestry and land use coalition were not part of official decision-making venues due to the primacy of ecological criteria in establishing the Natura 2000 network (I27-37).

Enforcement challenges became evident soon after the MS had to implement the Directive against increasing land users’ opposition across almost all MS. Related to forestry, this opposition resulted in neglect or inappropriate legal transpositions of the formal rules, delayed and insufficient designations of protected areas, and questionable conservation management. The Commission/DG ENV took legal actions against non-compliant MS. With almost 20 years delay, the formal area designations are now largely completed. The Natura 2000 network today constitutes more than 26,000 sites, half of which are located in and/or depend on forest ecosystems. This means that one fourth of all EU’s forests are now protected in the Natura 2000 network (D5-9; D14-15; I12-13).

The widespread resistance from land use actors led the environmental coalition to temporarily mute its top-down conservation biology implementation beliefs, and to engage in strategic cooperation with the forestry and land use coalition in the 2000s. National environmental authorities sought to work together with landowners, hunters, and concerned citizens within EU and MS expert working groups. This resulted, for instance, in voluntary Natura 2000 contracts with compensation payments in some MS. The top-down regulatory approach was, however, never fully abandoned, and repeated legal procedures enforced the protection of Natura 2000 sites in forests (Borrass et al. 2015; Sotirov et al. 2015).

A summary of the policy advocacy coalitions in the EU Natura 2000 policy for the period 1988–2016 and post 2018 is provided in Table 1.

#### Policy evaluation

From 2015 to 2016, the EU Nature Directives went through a policy evaluation process, called fitness check. Initiated by the then new Commission President Junker under the EU’s REFIT program—to make EU laws simpler and less costly—the Fitness Check concluded that the Nature Directives were fit for the purpose but would need better implementation. Hence, no substantial change of the policy framework was made (D26-28).

Coalitional politics during the fitness check were marked by significant cross-coalitional shifts (Table 2). The forestry and land use coalition, supported by economic development-friendly MS authorities and the Commission President Junker administrations, initiated the evaluation of the Nature Directives looking into a more flexible implementation approach to increase scope for bioeconomy businesses. However, the environmental coalition successfully blocked these attempts in the EU public consultation. Environmental NGOs published several policy papers and succeeded to ensure the political support of more than 500,000 European citizens through a #NatureAlert online campaign advocating no change. Moreover, a range of MS environmental ministers sent Commissioner Junker a joint official letter that MS will not support policy changes; instead, they requested improved implementation and more EU funding for Natura 2000. The Commission/DG ENV and the Parliament/ENVI Committee published studies concluding that the Nature Directives were still fit for their purpose (D22-23; D27-28).

**Table 2:** Shifting coalitions during the Fitness Check of the EU Natura 2000 policy, 2016–2018 (ESM, Tables S2, S3)

	Pro-change alliance	Contra-change alliance	
	Forest and land use bioeconomy coalition	Nature conservation coalition	Non-bioeconomy business coalition
Basic value priorities	Economic development > nature conservation	Economic development < nature conservation	Sustainable development
Entities whose welfare is of greatest concern	Industry, bio-economy, agriculture, forestry	Nature, biodiversity	Industry, economy, nature
Overall seriousness of the problem	The nature directives are a burden for economic development (expensive, time-consuming) Nature directives lead to legal uncertainties	The nature directives will be weakened by the review/fitness check threatening nature and biodiversity A merge of the nature directives will lead into a stagnancy over 5 to 6 years	Changes in the nature directives will lead to legal uncertainties for businesses A weakening of the nature directives will threat ecosystem services and thereby sustainable development
Basic causes of the problem	Strictness of the nature directives Too much bureaucracy	The intention of the European Commission (high political level) to revise the Nature Directives	The intention of the European Commission (high political level) to revise the Nature Directives
Problem solutions	Reform the EU nature directives	Do not change EU nature directives but improve implementation	Do not change the EU Nature Directives
Members	European Commission (High political level), bioeconomy businesses, land and forest owners (e.g., Business Europe, EUSTAFOR, CEPF, COPA-COGECA, ELO)	Environmental NGOs EU Environmental Council Several environmental ministers European Parliament, Commission/DG ENV	Some (bigger) non-bioeconomy business actors and their associations (e.g., CEMBUREAU, EWEA, UEPG, Eurelectric)

During that period, the environmental coalition formed a strategic alliance with a third policy coalition consisting of non-bioeconomy businesses such as wind power energy, electricity, cement and construction industries. This cross-coalitional “contra change” alliance argued for the intrinsic value of nature, like the environmental coalition. It further underlined that changes in the Nature Directives might lead to legal uncertainties for businesses, preventing sustainable development. Still, it requested flexible implementation from MS, like the forestry and land use coalition. This constellation eventually resulted in a defeat of the “pro-change” bioeconomy coalition of forestry and land use actors at the end of the Fitness Check (D17-18; D21; D29).

#### Recent policy development

The polarization between the environmental and the forestry and land use coalitions currently continues after the Commission launched a new EU Biodiversity Strategy to 2030 and a new EU Forest Strategy, setting more ambitious conservation targets and claiming a clearer EU forest decision-making power under the environmental competence, goals that meet with opposition by state forestry authorities in some forest-rich MS. Linking biodiversity conservation and climate change mitigation, the new EU Biodiversity Strategy aims to extend the existing Natura 2000 network of protected area, including stricter protection of primary and old-growth forests. While both strategies aim for more ambitious biodiversity conservation targets than in the past, their impact on forestry and conservation practices in the MS remains to be seen given recent heated debates surrounding them and their implementation (D15, D32).

Table 3 provides a summary overview of the traced Natura 2000 policy process.

**Table 3:** Process tracing of EU Natura 2000 policy development: summary overview

Phase	Coalitional politics	Institutional venues	Outcome
Agenda-setting, adoption and implementation	Adversarial politics: Environmental (Pro-change) vs. Forestry and Land Use Coalitions (Contra-change) (X_1_)	Environmental coalition dominates Commission (Z_1_), Council (Z_2_), and ECJ (Z_3_)	Policy change: adoption (Y_1a_) and legal enforcement of EU Nature Directives (Y_1b_)
Policy evaluation (Fitness Check)	Cooperative politics: Strategic Pro-CONTINUITY Alliance between Environmental and Non-bioeconomy Industry Coalitions (X_2_) Adversarial politics: Environmental (Contra-Change) vs. Forestry and Land Use Coalitions (Pro-Change) (X_1_)	Environmental Coalition and Strategic Alliance dominate Commission (Z_1_), Council (Z_2_) and public consultations (Z_3_) Forestry and Land Use Coalition dominate Commission President (Z_4_)	Policy continuity: no change of Nature Directives after the Fitness Check (Y_1c_)
Policy implementation I	Adversarial politics: Environmental vs. Forestry and Land Use Coalitions (X_1_)	Forestry and Land Use Coalition dominates member state executives and target groups; environmental coalition controls environmental authorities (Z_5_)	No or incremental policy change: Insufficient and protracted implementation (Y_2_)
Policy implementation II	Cooperative politics: Strategic cooperation between Environmental and Forestry and Land Use Coalitions (X_2_)	Mutual control of EU and national level decision-making venues and professional forums (Z_6_)	Mixed policy change: integration of land users’ values and interests in Natura 2000 management planning (Y_3_)

### FLEGT: the EU’s timber legality and anti deforestation policy

#### Policy initiation

Public awareness of tropical deforestation grew in the 1980s and 1990s, and illegal logging gained increased political attention after the G8 countries tackled the issue with their Action Programme on Forests in 1998 under the UK Presidency (D1). Similar to the Natura 2000 case, a coalition of environmental NGOs (ClientEarth, EIA, FERN, Greenpeace, WWF) formed and strategically framed illegal logging as an issue experienced abroad mostly in tropical countries, but connected to drivers operating “at home” in the EU marketing and consuming illegally sourced wood products (D3; I32-35).

As a response, the Commission and MS jointly adopted the EU’s FLEGT Action Plan in 2003, favoring voluntary “supply-side” economic incentives to tropical countries. This mainly included FLEGT voluntary partnership trade agreements (VPAs) with the EU (negotiated by the European Commission on behalf of the EU countries), requesting successful multi-stakeholder negotiations about good governance and national timber legality definitions, as well as establishing timber tracking systems in tropical countries in exchange for preferred EU market access for tropical wood and wood products from the respective partner country (I82-83).

Subsequently, environmental NGOs, but also some countries and specifically UK pushed for additional policy measures. For instance, in 2004, 180 NGOs, scientists and citizens from Europe, Russia and Asia, led by Greenpeace, signed a joint statement requesting new EU legislation to “outlaw imports of all illegally sourced timber products into the EU market”. Environmental groups also stated that the “issue of forest sustainability needs to be addressed” since “legal and illegal logging are often closely linked and (…)legal logging can be highly destructive”, and because the forest industry would be “unable to regulate itself and is destroying forests and peoples' livelihoods on a grand scale” (D26). Later, NGOs worked together with European timber companies and retailers (B&Q, IKEA, Castorama, OBI) to sign a series of FLEGT industry statements calling EU institutions and Member States to adopt a new EU law to make it “illegal to import all illegally sourced timber and wood products into the European market”, while stimulating fair competition and sustainable markets (D4).

In response, the Commission (DG ENV) carried out assessments and consultations on future EU policy options against illegal logging, which showed that the bilateral VPA negotiations were slower than expected, and that further EU legislative actions were needed to strengthen the policy against illegal logging (D1). In September 2008, the governments of UK, the Netherlands and Denmark, supported by Austria, Bulgaria, Germany, Romania, and Luxembourg, requested the Commission to publish a communication on additional legal options, including a proposal for EU legislation (D2, D3).

#### Policy adoption

In October 2008, the Commission presented a first EU legislative proposal emphasizing industry self-regulation through a due diligence approach of risk management along timber supply chains (C2, C3). The Commission stressed its intention to simplify the requirements for economic operators and national authorities while stimulating economic competitiveness (D1). In response, NGOs and the environmentally oriented branches of the Commission (DG ENV, DG DEVCO) built a cross-coalitional alliance with timber importing industries, big multinational timber retail companies and national authorities from forest-poor timber importing MS (the UK, Netherlands, Denmark, Belgium) to demand a stricter EU Timber Regulation (EUTR) that would ban illegally sourced timber from entering the EU market (prohibition). Interestingly, this cross-coalitional alliance did not advocate for additional mandatory sustainability rules, as initially requested by NGOs. This mirrored a strategic concession from the environmental groups toward their industry partners. The industry groups, on their part, saw the strategic collaboration with NGOs as a chance to revert the image of tropical timber, portrayed by NGOs and scientists to be of risky or criminal origin (I1-4; I32-36; I69-72; D3-5; D27-29).

A forest sector coalition composed of domestic forestry interest groups, national forestry state authorities from forest-rich export-oriented countries (Austria, Germany, Sweden, Finland, East European countries) and economically oriented branches of the Commission (DG TRADE and DG AGRI) mobilized to oppose to the EUTR in general, and to a prohibition clause in particular. They rejected any EU legal measures on the demand side that could affect businesses, and opposed creating a precedent of EU environmental competency in forestry matters that would constrain rights to commercially use forests and timber resources. They argued that the European forest sector was fully committed to sustainable forest management, and requested the use of supply-side measures (FLEGT VPAs), voluntary industry self-regulation (codes of conduct) and market incentives (forest certification) to tackle the “outside” illegal logging problem (D10-11, D25, D31; I8-15; I45-51; I57-64).

A subsequent Commission’s proposal for a stronger EUTR with a prohibition clause became subject to heated political debates in Council meetings and the Parliament from 2008 onwards. Despite the resistance of the forest sector coalition members, the Council, the European Parliament and the Commission finally adopted the EUTR in July 2010 after three informal trialogue meetings and two years of tough political negotiations. The Parliament had threatened to not finalize the legislative process without inclusion of a prohibition clause, and to extend the scope of the regulation toward mandatory forest sustainability standards. Subsequently, the EUTR-supportive Spanish and Belgian governments, holding the Council Presidencies in 2010, brokered a political compromise between the Parliament, the Council and the Commission in that the prohibition would be the price of a deal to not include sustainability standards, and also emphasizing that the prohibition would have high symbolic importance (I1-4; I7; I8-12; I30-31).

The European Parliament passed the EUTR with 644 votes in favor, 25 votes against and 16 abstentions. In the final vote in the Council under the Presidency of Belgium in October 2010, all MS voted for, with only Sweden voting against, with the Czech Republic and Portugal abstaining (Sotirov et al. 2017).

The adopted legal text of the EUTR contains a legal ban against placing illegally sourced wood and wood products on the European market. It also requests economic operators to exercise due diligence to secure timber legality along international supply chains. It does not contain any legal obligation to meet sustainability standards (D30).

A summary of the policy advocacy coalitions and shifting coalitions in the EU FLEGT/EUTR policy for the period 2004–2020 is provided in Table 4.

**Table 4:** Advocacy coalitions and shifting coalitions in the EU FLEGT/EUTR Policy 2004–2020 (I1-86)

	Contra-change coalition	Pro-change alliance	
	Traditional forest sector coalition	Environmental coalition	Progressive forest sector coalition
Basic value priorities	Economic development > environmental sustainability	Economic development < environmental sustainability	Sustainable economic development (Economic development = environmental sustainability)
Entities whose welfare is of greatest concern	Industry, Economy, Agriculture, Forestry	Nature, Biodiversity, Social rights	Industry, Economy, Nature, Public Welfare
Overall seriousness of the problem	Illegal logging as important problem, but also as a problem that originates outside Europe and needs to be tackled there	Illegal logging as a basic problem for forest governance globally; given the interconnectedness of forest product chains and environmental destruction a strong European response is needed	
Basic causes of the problem			
Problem solutions	Tropical exporting countries need to solve the problem, it is not the problem of European forest owners and companies. Voluntary measures and industry self-regulation should be encouraged to tackle the problem and remain WTO conform. Otherwise overregulation and administrative burdens are created for the forest industry, and hard legislation is hardly implementable	European markets need to be closed for illegally harvested wood Voluntary measures are not enough, legislation is needed to make sure that illegally harvested wood remains outside the market “Hard regulation” with prohibition is an opportunity to recover the image of the (timber importing) forest industry	
Members	State forest authorities from forest-rich countries with strong domestic forest industries (Austria, Czech Republic, France, Finland, Germany, Italy, Hungary, Latvia, Poland, Romania, Slovakia, Sweden) State and non-state forest owners Domestic timber producing industries European Commission/DG Trade and DG Agri	Environmental NGOs, think tanks and scientists, Commission/DG ENV, and EU Parliament/COMENVI	Timber traders, importers and big retailers State forest authorities from forest-poor countries with timber importing economies and strong environmental NGOs (Belgium, Denmark, Netherlands, Spain, UK)

#### Policy implementation, evaluation, and recent developments

Similar to EU Natura 2000 policy, the domestic implementation of the EUTR has been slow, and an overall lack of enforcement is evident in several MS. During its domestic implementation, coalitions and strategic alliances have partly continued to shift, and partly ceased, while the ideological confrontation between environmental and industry actors relating to sustainability vs. legality remains (I4; I19-29; I34-44; I63-81; McDermott and Sotirov 2018).

Stakeholder feedback for the 2020 Fitness Check of the EUTR indicates consensus among both NGOs and forest industry actors on the main purpose of the EUTR to fight illegal logging worldwide. However, NGOs, the Commission (DG ENV), the Parliament (COMENVI) and forest-poor MS (Netherlands, Denmark, Belgium) expressed the need to complement the EUTR with a new EU legal instrument to control forest-risk agricultural supply chains to help achieving biodiversity conservation and climate protection goals. Forest industry actors in the forest-poor countries (dependent on imports and a good reputation of tropical timber) remained concerned about market disadvantages due to the incoherent implementation of the EUTR (D32-34).

Recently, the Commission/DG ENV and the Parliament/ENVI Committee started formulating a new EU legal framework for action against (global) deforestation and forest degradation. Building on the EUTR, this currently evolving legal framework suggests mandatory due diligence rules for forest-risk agricultural (soy, beef, palm oil) and timber commodities that are placed on the EU market. Beyond legality verification, this may also include sustainability standards relating to environmental and human rights protection. This new legal framework would respond to the fact that nearly 80% of tropical deforestation is related to agricultural expansion in response to global food demand, including EU consumption. Nevertheless, the ideological framing division of timber legality versus forest sustainability remains also in this recent debate (D34).

Table 5 provides a summary overview of the traced FLEGT (EUTR) policy process.

**Table 5:** Process tracing of EU FLEGT/EUTR policy development: summary overview (ESM, S3, S4)

Phase	Coalitional politics	Institutional venues	Outcome
Agenda-setting and policy negotiations	Adversarial politics: Environmental (Pro-change) vs. Forest Sector (Contra-change) Coalitions (X_1_)	Environmental coalition dominates Commission (Z_1_) and Parliament (Z_3_); Forest Sector Coalitions dominates Council (Z_2_)	Policy stalemate: EUTR prohibition debate (Y_1b_)
Policy adoption	Cooperative politics: Strategic Pro-CHANGE Alliance between Environmental and Progressive Forest Sector Coalitions (X_2_)	Strategic Pro-Change Alliance eventually dominates Commission (Z_1_), Council (Z_2_) and Parliament (Z_3_)	Policy change: adoption of the EU Timber Regulation (Y_1_)
Policy implementation, Fitness Check EU FLEGT (EUTR), and policy development	Adversarial politics (X_1_) and strategic cooperation (X_2_) between Environmental and Forest Sector Coalitions	Forest Sector Coalitions dominate member state authorities (Z_5_) and target groups (Z_6_); environmental coalition controls Commission (Z_1_)	Mixed policy change: Policy continuity with EUTR (Y_1a_) and inconsistent implementation across EU countries (Y_1b_); Policy change with a new EU bill on forest-risk commodities (Y_2_)

### LULUCF: EU forest climate policy

#### Policy initiation

Until ca. 2010, EU institutions and member states hesitated to include forestry and agriculture in climate policy, as this was regarded to be too complex, uncertain, and difficult to regulate (Ellisson et al. 2014; Savaresi et al 2020). In preparations for the UNFCCC COP 15 in Copenhagen 2009, the Council adopted an agenda for EU action on climate accounting from the LULUCF sector. In October 2014, the European Council decided that the LULUCF sector should be considered in EU climate policy. The Commission drafted a legal proposal in July 2016. It used scientific data to show that the LULUCF sector was key to consider in EU climate policy since it covered more than three-quarters of the EU’s territory, held large stocks of carbon, and absorbed the equivalent of nearly 10% of total EU GHG emissions each year (D1-4, D7).

#### Policy adoption

The Council and the Parliament took their final decision to adopt the EU LULUCF Regulation in May 2018. Few compromises paved the way for policy adoption, granting, however, significant national flexibility to MS. These changes included, inter alia, the determination of a Forest Reference Level (FLR) as a benchmark to assess future carbon sink efforts from forestry and land use in the 2021–2030 period based on a projected continuation of traditional forest management practices from 2000 to 2009 (instead of 1990 to 2009), and not penalizing emissions from forest aging and climate-induced forest damages (drought, fires, storms, pests and diseases). The Regulation also acknowledges the climate benefits of (intensive) forest use and wood products, which can store carbon and substitute for emission-intensive materials (I1-3; I5-6; I7-14; D9-10; D13, D17-18).

At its core, the LULUCF regulation obliges MS to ensure that accounted GHG emissions from the LULUCF sector are balanced by at least an equivalent accounted removal of CO_2_ from the atmosphere, known as the “no debt rule”. In line with this, the regulation establishes a legally binding EU policy for how MS should measure emissions and removals from management practices in their forests. This decision addresses earlier criticism from environmental groups that emissions from biomass in energy production were not accounted for under previous EU policy, e.g., the Renewable Energy Directive—RED I and RED II (D19).

The final policy outcome reflected a mixture of the beliefs of three policy advocacy coalitions. A first coalition formed to secure what they believed to be the environmental integrity of the LULUCF Regulation. This coalition included environmental and climate NGOs (e.g., FERN, WWF, and BirdLife), conservation biology and climate protection scientists, as well as the Commission (DG ENV) and the Parliament (COMENVI), accompanied by some MS environmental and nature protection public authorities. Initially several coalition members opposed the inclusion of the LULUCF sector in EU climate policy as they feared that this may dilute climate policy efforts. They were open to inclusion of the LULUCF in the EU reduction commitments only on the condition that there would be separate mandatory carbon reduction targets, full accounting for leakage or “hidden emissions”, including from bioenergy, accounting of harvested wood products that would address uncertainties, and forest management accounting based on the historical period of 1990–2012. They also advocated for legally binding EU rules safeguarding that carbon is enhanced in standing (mature and old growth) forests while promoting environmental integrity in forest management, protected forest areas, and ecological restoration (I1; I28-32; I16-18; I33-35; D5, D8, D14-17).

The environmental integrity coalition was pitted against a more influential forest bioeconomy coalition. The latter was not only composed of forestry sector actors and forestry scientists at the EU and national levels (e.g., CEPF, EUSTAFOR, CEPI). A range of national public forestry authorities representing forest-rich countries with economically important national forest sectors (Finland, Estonia, Poland, Romania, partly France and Sweden) as well as like-minded branches of EU institutions such as the Commission (DG AGRI) and the Parliament (COMAGRI) were also active members in this forest bioeconomy coalition. This coalition supported timber-use-oriented forestry and material/energy substitution with wood-based products, strategically framed as positive climate mitigation action. They also pressed for national level decision-making flexibility in implementing the EU’s LULUCF accounting rules. They called for national forest sector strategies, as well as for forest reference levels (FRLs) based on historical forest use, and communicated studies that not harvesting timber would enhance risks that carbon stored over decades in forests is released due to climate change driven forest disasters (I4; I7-13; I19-25; D6, D11, D17).

The Commission (DG CLIMA) and national public authorities in countries where forestry and environmental interests were similarly powerful (Germany, France, and partly Sweden) or where climate-environmental interests overrode forestry interests (Italy, Spain) formed a third, pro-climate EU regulation policy coalition. This coalition skilfully merged the (polarized) positions of the other two coalitions. On the one hand, they accepted that the environmental integrity coalition was not taking the diversity of national forest situations into account, and hence supported some national flexibility in line with the claims of the forest bioeconomy coalition. On the other hand, they criticized the forest bioeconomy coalition for being too slack regarding environmental integrity, and hence supported a mandatory EU LULUCF accounting approach with some environmental safeguards and decision-making by the Commission (I2-3; I9-14; I10-16).

The climate policy coalition could eventually find a compromise between the other coalitions during the negotiations and final adoption of the LULUCF Regulation between 2016–2018. They built a strategic cross-coalitional alliance with national agricultural authorities and interest groups in forest-extensive but agriculture-intensive EU countries (the UK, Ireland, Denmark, Belgium, the Netherlands). These agricultural actors considered the Commission’s legal proposal as an opportunity to compensate for emissions from the agriculture sector through carbon offsets either in their own forests (that play more of an ecological than economic role) or in other MS’ forests (D9-10; D17-19; I2-3; I9-14).

The three policy advocacy coalitions were not entirely stable during the negotiation and adoption process of the LULUCF regulation. For instance, a shift in the French, German and Swedish positions from a contra-regulatory/pro-forest bioeconomy to a pro-climate regulatory position followed from political changes in governments with new commitments to act against climate change, along with the policy spill-over impacts from the LULUCF commitments in the Paris Agreement in 2015. France and Germany played a major role in finding a compromise, while Finland and many Eastern European countries remained quite stable in their pro-forest bioeconomy and national flexibility beliefs (I7-14).

A summary of the policy advocacy coalitions and shifting alliances (beliefs, participants) is provided in Table 6.

**Table 6:** Policy advocacy coalitions and shifting coalitions in the EU LULUCF Policy, 2010–2018 (I1-38)

Environmental integrity coalition	Forest bioeconomy coalition	Strategic PRO-Regulation alliance	
		Climate coalition	Agricultural coalition
Policy core beliefs			
Problem definition			
Foresters misunderstand the real problem of climate change and the negative role of forestry therein Little or no timber harvesting enhances carbon stocks in standing forests and land use, protects forest biodiversity and secures sustainable management of forest	Environmentalists misunderstand the real problem of climate change and the positive role of forestry therein. Timber harvesting reduces the risk to see carbon stored during decades in forest being suddenly released because of climate change risks	Extreme environmental and forestry positions: the environmental integrity view ignores national forest specificities, whereas forestry is permissive to environmental integrity Climate change, forest bioeconomy and environmental integrity are possible and complementary	Intensive land use (farming) is being both a victim and part of the solution to climate change risks
Policy solution			
Opposition to EU LULUCF Regulation (No-Change): countries shouldn’t have GHG emissions flexibilities and credits to compensate their losses Support only when environmental integrity with biodiversity safeguards are secured	National flexibilities and national forest strategies are essential. The benefit of material substitution with wood-based products should be recognized in mitigating climate change	Support for EU LULUCF regulation (Pro-Change). Need for a compromise between the different parties	Support for EU LULUCF Regulation (Pro-Change); National and EU-wide flexibility to allow forests to offset agricultural emissions
Members			
Environmental NGOs (Fern, FNE, CAN, BirdLife, Naturschutzbund) Conservation biology and climate protection sciences EU Commission (DG ENV) European Parliament (ENV Committee)	Forest sector interest groups (CEPF, CEPI, EUSTAFOR, AGDW, Fransylva) National forestry authorities in forest-rich and forest sector powerful member states (Finland, Estonia, Poland, Romania, partly France and Sweden) Forest scientists EU Commission (DG Agriculture and Rural Development) EU Agricultural Council European Parliament (Agriculture Committee-COMAGRI)	EU Commission (DG CLIMA) National environmental authorities in forest- rich member states with both economic and environmental priorities (Germany, France, Sweden, Italy, Spain, Czech Republic and Slovakia) Council of EU Environmental Ministers	National agricultural and environmental authorities in forestry extensive but agriculture-intensive member states (Denmark, UK, Ireland, the Netherlands, Belgium and for some issues Germany) National and EU farmer interest groups

#### Policy implementation

Between late 2019 and 2020, the Commission developed an overarching EU climate neutrality and climate smart economy policy with the adoption of the European Green Deal and the 2030 Climate Target Plan. This EU climate policy aims to cut GHG emissions by at least 55% by 2030, and adopts a legally binding commitment to make the EU-27 climate neutral by 2050. Therein, the Commission and MS recognize the importance of forests in achieving climate goals and request stepping up policy, legal and practical climate action in the LULUCF sector (D20-22).

In October 2020, the Commission amended the existing LULUCF legislation with a delegated act setting FRLs that each country must apply between 2021 and 2025. It remains to be seen, however, how national implementation of the LULUCF regulation will progress given complex technical rules and recently enhanced timber removals partially related to forest disturbances reducing forest sinks in several EU countries (Table 7).

**Table 7:** Process tracing of the EU LULUCF policy development: summary overview (ESM, S5, S6)

Phase	Coalitional politics	Institutional venues	Outcome
Agenda-setting and policy negotiation	Adversarial politics: Environmental vs. Forest Bioeconomy Coalitions (X_1_)	Forest Bioeconomy Coalitions partly controls Council (Z_2_); Environmental coalition dominates Parliament (Z_3_)	Policy stalemate: EU LULUCF controversy (Y_2_)
Policy adoption and policy development	Cooperative politics: Strategic Pro-CHANGE Alliance between Climate and Traditional Agricultural Coalitions (X_2a_); Climate coalition integrates and tries to compromise between Environmental and Forest Bioeconomy Coalitions’ beliefs (X_2b_)	Climate Coalition and Strategic Alliance dominate Commission (Z_1_) and Council (Z_2_), and eventually control Parliament (Z_3_)	Policy change: adoption of the EU LULUCF Regulation (Y_1a_), Policy Continuity: enhanced LULUCF under the EU Green Deal (Y_1b_)

## Discussion and conclusions

How can we explain both change and continuity in EU environmental forest policy over time from a coalitional politics perspective?

The empirical evidence from all three cases supports the “Adversarial coalitional politics” Hypothesis 1 in that EU environmental forest policy is characterized by a *stable* line-up of competing advocacy coalitions—and namely a forestry and an environment coalition—that differ fundamentally regarding their core beliefs. These coalitions have strongly influenced the policy debates and outcomes of EU environmental forest policy in all three issue areas. The ideological divergence between environmental and forest use policy advocacy coalitions is evident not only among MS authorities, but also within the EU institutions.

Specifically, our findings show that the environmental coalition consists of environmental NGOs who tend to liaise both with each other and like-minded EU institutions (Commission/DG ENV, Parliament/COMENVI; Council ENVI) and with national environmental authorities that are strong domestically specifically in forest-poor countries with weak forest industries and strong NGOs (Belgium, Denmark, Netherlands, Luxembourg, UK). They share normative beliefs concerning biodiversity conservation and support tackling global sustainability challenges. They advocate environmentally oriented regulatory EU polices to advance their beliefs, and oppose MS flexibility and non-legally binding policies giving leeway to commercial use of forests. Southern EU MS (e.g., Greece, Italy, Spain) tend to share similar environmental core beliefs, but support stronger EU forest environmental policy also for the expectation of EU financial support to tackle issues of rural land abandonment, non-profitable forestry and climate change problems such as droughts and fires (Winkel and Sotirov 2016; Frei et al. 2020).

Reversely, a coalition of forest owner organizations and forest industries, state forestry authorities in forest-rich countries with strong domestic forest industries (mainly Sweden and Finland, but often also Austria, France, Germany and Eastern European countries) tend to liaise with like-minded EU institutions (Commission/DG AGRI and DG Trade, Parliament/COMAGRI, Council AGRI). This forest use coalition tends to oppose common EU forest policy, especially if it would be based under an environmental competency. Instead, it supports active forestry, and prefers flexible forest-economy friendly policies under MS competency.

These findings mirror similar insights for a large set of policy studies (see Sotirov and Memmler 2012; Jenkins-Smith et al. 2014 for the ACF, Eckerberg and Sandström 2013 and Sandström et al. 2013 for forest conflicts) showing the importance of a fundamental ideological divide between environmental (nature conservation) and economic (land use) core beliefs for environmental and natural resource policy making.

The ideological divide goes along with another observation from our three case studies that the overall coalitional setting of EU environmental forest policy is quite stable unless a coalition skilfully exploits new opportunities for a policy reform (Hypothesis 2) or engages in a cross-ideological strategic alliance building (Hypothesis 3). Concretely, the first case could be observed for the agenda setting and policy adoption phase of the Habitats Directive in the early 1990s: Here, the success of the nature protection coalition was connected to their ability to strategically use opportunities of the EU system (Council and Commission venues in the EU-12), spillovers from international biodiversity policy, new conservation science evidence as well as changes in governments to translate its conservation beliefs into the EU Nature Directives. Later on, when the two new EU environmental forest policies (FLEGT and LULUCF) emerged, such strong influence of the environmental coalition was no longer observed.

Hence, except for the adoption phase of the Habitats Directive, our findings support Hypothesis 3 according to which strategic alliances occur across different advocacy coalitions and are a precondition for policy change. These cross-coalitional alliances—also labeled Baptist and Bootlegger (B&B) coalitions in the political economy literature (Yandle 1983)—were built by environmental NGOs and parts of the business sector, for instance non-bioeconomy businesses (Natura 2000 Fitness Check), timber importing industries and retailers (FLEGT/EUTR), or agricultural interests (LULUCF). They were decisive to achieve policy change (FLEGT/EUTR, LULUCF)—or prevent it, as in the case of the evaluation of EU nature conservation policy (Natura 2000).

Finally, all three case studies (except to some degree the Natura 2000 adoption phase) confirm our fourth hypothesis in that changes in EU environmental forest policy will entail a mixture of core beliefs from various coalitions as outcome of strategic alliance building and compromises that were necessary to achieve policy change. In sum, with the help of such alliances and despite the opposition of the forestry and land use coalition, the environmental coalition was able to establish EU environmental forest policies in all three investigated issue areas.

What remains to be further explored, beyond the scope of this paper, is how such a gradual success of the environmental coalition at the EU policy level is counterbalanced by reluctant implementation at the level of MS. Existing research shows that dissatisfied MS and interest groups from the forestry and land use coalitions have been partly able to revert successes of the environmental coalition through weakening practical implementation and mitigating policy impact on the ground (see for instance Winkel et al. 2015). Here, a power game appears on a continuum between “how far MS can deviate” and “when do EU institutions dare to intervene” (Borrass et al. 2015).

In conclusion, our results can be summarized in a way that EU’s environmental forest policy is characterized by (i.) a stable long-term line-up of rivaling policy advocacy coalitions that are characterized by fundamentally different policy core beliefs and related (institutional) interests—with an environmental vis a vis a forest use coalition being most central; (ii.) dynamic processes of forming strategic alliances across different policy advocacy coalitions in relation to specific policy issues that evolve over time; and (iii.) changes in EU’s environmental forest policy that reflect the multi-faceted translation of policy core beliefs of involved actors’ coalitions.

In a forward-looking perspective, there is little doubt that cross-coalitional conflicts will endure in EU environmental forest policy and possibly intensify (Wolfslehner et al. 2020). An interesting question is in how far the EU’s ambition to be a leader in global sustainable development, as exemplified with the EU Green Deal, and the urgency of global policy issues such as climate change mitigation and adaptation, biodiversity conservation, and the interrelation between trade and sustainability issues, might affect the EU forest policy arena. The desire (or necessity) for climate policy leadership may overrun the special sector conflicts between the forest and the environmental community, resulting in unexpected defeats for one side or enforcing collaboration across the sectors. Previous research has, however, shown that climate change and a related “forest crises” might also intensify ideological polarization at the European environmental forest frontier. This is the case when problem perceptions, responsibilities and corresponding solutions are fundamentally different (De Koning et al. 2014). To investigate the impact of climate change and climate policies on EU forest policy will hence be an exciting issue for future research.

## Supplementary Information

Below is the link to the electronic supplementary material.

Supplementary file 1 (PDF 883 kb)
