# Supplementary material for: The coalitional politics of the European Union’s environmental forest policy: Biodiversity conservation, timber legality, and climate protection
Source: Ambio. 2021 Oct 10;50(12):2153–67. doi: 10.1007/s13280-021-01644-5 (PMC8563925; doi:10.1007/s13280-021-01644-5)
Supplement: Supplementary file 1 — (PDF 883 kb) [file 13280_2021_1644_MOESM1_ESM.pdf]

Ambio

Electronic Supplementary Material

This supplementary material has not been peer reviewed.

Title: **The Coalitional Politics of the European Union's Regulatory Environmental Forest Policy: Biodiversity Conservation, Timber Legality, and Climate Protection**

Authors: (to be specified after acception)

Table S1: List of key informant interviews, EU nature protection and biodiversity policy, 2011-2018

Interviewees' affiliation	Number	Abbreviation used in text
Council of European Union, Directorate for Environment<br>Council of European Union, Directorate for Agriculture and Forestry	2<br>1	I1-2<br>I3
European Parliament, COM-ENVI (1x conservative, 1x social-democrat, 1x green party)	3	I4-6
European Commission, DG Environment, Natura 2000 Unit<br>European Commission, DG Environment, Agriculture and Forest Unit<br>European Commission, DG Environment, Legal Enforcement Unit<br>European Commission, DG Agriculture and Rural Development,<br>Forestry Unit European Environmental Agency, Biodiversity Unit, Forest and Environment	3<br>2<br>2<br>1<br>2	I7-9<br>I10-11<br>I12-13<br>I14<br>I15-16
European and national environmental NGOs (BirdLife, EEB, FERN, Greenpeace, IUCN, WWF)	10	I17-26
European associations of national private forest owner organisations (CEPF)<br>European association of national state forest enterprises (EUSTAFOR<br>European association of national landowners and farmers organisations (ELO, Copa-Cogeca)<br>European associations of forest industries (Cei-Bois, CEPI)<br>European federation of national hunters associations (FACE)	2<br>2<br>4<br>2<br>1	I27-28<br>I29-30<br>I31-34<br>I35-36<br>I37
State forestry and environmental authorities of Member States (Austria, Bulgaria, France, Croatia, Germany, Italy, Netherlands, Spain, Slovakia , Sweden, UK)	20	I38-57
Scientists and think tanks in the field of conservation biology and forestry (IUFRO, JRC, ETC-BD, EFI)	5	I58-63
**Total:**	63	

Table S2: Policy documents, EU biodiversity policy 1988-2020, subject to content analysis

**Document Type**	**Year of Publication**	**Author, Institution**	**Title (Abbreviation used in the main text)**
Directive	1988	European Commission	Proposal for a COUNCIL DIRECTIVE on the conservation of natural and semi-natural habitats and of wild fauna and flora (D1)
Directive	1990	European Commission	SUPPLEMENTARY ANNEXES TO THE Proposal for a COUNCIL DIRECTIVE on the protection of natural and semi-natural habitats and of wild fauna and flora (D2)
Directive	1991	European Commission	Modified proposal for a COUNCIL DIRECTIVE on the conservation of natural and semi-natural habitats and of wild fauna and flora (D3)
Directive	1992	Council of the European Communities	Council Directive 92/43/ECC of 21 May 1992 on the conservation of natural habitats and of wild flora and fauna (Habitat Directive) (D4)
Guidelines	2000	European Commission	Managing Natura 2000 sites: The provisions of Article 6 of the 'Habitats' Directive 92/43/EEC (D5)
Guidelines	2003	European Commission	Natura 2000 and forests ‘Challenges and Opportunities’. Interpretation guide (D6)
Report	2004	European Commission	Report from the Commission on the implementation of the Directive 92/43/EEC on the conservation of natural habitats and of wild fauna and flora (D7)
Booklet	2006	European Commission / European Court of Justice	Nature and Biodiversity. Ruling of the European Court of Justice. Legally binding case law (D8)
Guidelines	2007	European Commission	Guidance document on Article 6(4) of the 'Habitats Directive' 92/43/EEC (D8)
Technical Report	2007	European Commission	Interpretation Manual of European Union Habitats (D9)
Directive	2007	Council of the European Union	Council Directive 92/43/ECC of 21 May 1992 on the conservation of natural habitats and of wild flora and fauna (Habitat Directive) *(consolidated version)* (D10)
Technical report	2008	European Commission	Management of Natura 2000 Habitats *Luzulo-Fagetum* beech forests 9110 (D11)
Report	2009	European Commission	Composite Report on the Conservation Status of Habitat Types and Species as required under Article 17 of the Habitats Directive (D12)

Guidelines	2011	European Commission	Guidelines on Natura 2000 and forestry (D13)
Guidelines	2015	European Commission	Guidance on Natura 2000 and forests, incl. Frequently asked questions on Natura 2000 and Forests (Part I-II) and Good practice experiences from different Member States in managing forests in Natura 2000 (Part III) (D14)<br>https://ec.europa.eu/environment/nature/natura2000/management/guidance_en.htm
Policy strategy	2011	European Commission	COMMUNICATION FROM THE COMMISSION TO THE EUROPEAN PARLIAMENT, THE COUNCIL, THE ECONOMIC AND SOCIAL COMMITTEE AND THE COMMITTEE OF THE REGIONS Our life insurance, our natural capital: an EU biodiversity strategy to 2020 COMMUNICATION FROM THE COMMISSION TO THE EUROPEAN PARLIAMENT, THE COUNCIL, THE ECONOMIC AND SOCIAL COMMITTEE AND THE COMMITTEE OF THE REGIONS Our life insurance, our natural capital: an EU biodiversity strategy to 2020/* COM/2011/0244 final */ (D15)
Policy Paper	2013	WWF, EEB, and BirdLife International	'Joint Letter to the Heads of State or Government', 22 October 2013 (D16), URL: http://www.birdlife.org/sites/default/files/attachments/Letter_HoG_Refit_Oct_22_2013_2.pdf.
Policy paper	2014	Renewable Grid Initiative (RGI)	'Open Letter to President-Elect of the European Commission Jean-Claude Juncker: The Need for Clear, Stable Nature and Climate Mandates for Grid Investments', 29 September 2014 (D17).<br>https://renewables-grid.eu/fileadmin/user_upload/Files_RGI/RGI_Publications/Position_Papers/RGI_Open_letter_Jean-Claude_Juncker.pdf.
Position paper	2015	FACE (Confederation of European Hunters Associations)	'FACE Nimmt Stellung Zum Fitness-Check: Warum FACE Die Vogel- Und Habitat-Richtlinien Unterstützt' (D18).<br>https://web.archive.org/web/20160418000626/http://face.eu/sites/default/files/documents/german/face_statement_fitness_check_de_2.pdf.
Position Paper	2015	CEPF	Position Paper. Fitness Check of the EU nature legislation (D19).<br>URL: http://www.cepf-eu.org/vedl/CEPF%20position%20paper_Fitness%20C

			heck%20of%20Birds%20and%20Habitats%20Directives_Nov_2015(1).pdf
Position paper	2015	CEPF (Confederation of European Forest Owners), Copa-Cogeca (European framers and agri-cooperarives), ELO (European Landowner Organization), and USSE (Union of Foresters in Southern Europe).	'Joint Letter by CEPF, Copa-Cogeca, ELO and USSE to Commissioner for Environment Karmenu Vella', 17 July 2015. (D20)<br>https://www.cepf-eu.org/sites/default/files/document/joint_letter_REFIT_EU_nature_legislation_2015_07_17.pdf.
Position Paper	2015	BirdLife International & CEMBUREAU (European Cement Industry Association)	'Birds & Habitats Directives: Enhanced Implementation to Benefit Biodiversity' (D21)<br>URL: http://www.birdlife.org/sites/default/files/attachments/cembureau-birdlife_final_position_paper_birds_habitats_directive_2015-10-28_2_1.pdf.
Policy paper	2015	Barbara Hendricks, German Federal Minister of Environment	Letter to European Commission President Juncker (D22)<br>http://www.birdlife.org/sites/default/files/attachments/20140929_letterGEtoJuncker.pdf
Policy apper	2015	Environmental Ministers Europe (Germany, France, Luxembourg, Romania, Spain, Croatia, Poland, Italy, Slovenia)	Letter to European Environmental Commissioner Vella (D23).<br>https://www.bmu.de/fileadmin/Daten_BMU/Download_PDF/Strategien_Bilanzen_Gesetze/refit_joint_letter_en_bf.pdf
Position paper	2016	EUSTAFOR (European State Forest Association)	'Position Paper on the Fitness Check of the Birds and Habitats Directives and Their Implementation in State Forests'. (blog). 8 July 2016 (D24)

			https://eustafor.eu.
Position paper	2016	Europêche (Associations of national organizations of fishing enterprises in the EU) and EAPO (European Association of Fish Producers Organisations)	'Fishing Industry Disappointed over European Commission Decision to Maintain the Birds and Habitats Directives Unchanged'. (D25)<br><br>URL: http://europeche.chil.me/attachment/417eda96-08ae-4fa7-92a1-2762080b892e.
Policy document	2016	European Commission	Fitness Check of Nature legislation: Commission considers options for improved implementation of the Birds and Habitats Directives (D26).<br><br>URL (14.01.2017): http://europa.eu/rapid/press-release_MEX-16-4308_en.htm
Policy study and final report	2015, 2016	Milieu, IEEP (Institute of European Environmental Policy (IEEP), and ICF	'Fitness Check of the Birds and Habitats Directives'. 2016. (D27)<br><br>URL: https://ieep.eu/publications/fitness-check-of-the-birds-and-habitats-directives.<br><br>Evaluation Study to support the Fitness Check of the Birds and Habitats Directives. Technical report, Milieu, IEEP and ICF (D28)
Public consultation / stakeholder responses	2016	European Commission	EU Nature Directives 2020 'Fitness Check - Responses to the Evidence Gathering Questionnaire'. (D29)<br><br>https://ec.europa.eu/environment/nature/legislation/fitness_check/evidence_gathering/index_en.htm.
Policy campaigns	2016, 2017	BirdLife Europe, Friends of the Earth Europe, WWF EU, European Environmental Bureau	Nature Alert: Nature dodges a bullet: EU citizens save nature laws! (D30)<br><br>URL (18.1.2017): http://www.birdlife.org/campaign/NatureAlertAction<br><br>Nature dodges bullet: huge public campaign saves EU nature laws. URL(18.01.2017) (D31)<br><br>http://www.birdlife.org/sites/default/files/attachments/naturealertjointngopress-release.07.12.2016.pdf
Policy strategy	2020	European Commission	COMMUNICATION FROM THE COMMISSION TO THE EUROPEAN PARLIAMENT, THE COUNCIL, THE EUROPEAN ECONOMIC AND SOCIAL COMMITTEE AND THE COMMITTEE OF THE REGIONS EU Biodiversity Strategy for 2030 Bringing nature back into our lives. COM/2020/380 final (D32)

Table S3: List of key informant interviews, EU FLEGT/EUTR policy, 2013-2020

Interviewee's affiliation	Number	Abbreviation used in text
European Commission, DG Environment<br>European Commission, DG Industry<br>European Commission, DG Development Cooperation	4<br>1<br>1	I1-4<br>I5<br>I6
Council of the European Union, Council Working Group on Forestry<br>National forest and environmental authorities of EU Member States (Austria, Bulgaria, France, Finland, Germany, Netherlands, Italy, Romania, Spain, Sweden, UK)	1<br>22	I7<br>I8-29
European Parliament, COM-ENVI (1x social democrat and 1x green party)	2	I30-31
European and national environmental non-governmental organizations/NGOs (Client Earth, FERN, Greenpeace, WWF)	13	I32-44
State forestry enterprises and non-state forest owner organisations, and their European associations (EUSTAFOR, CEPF)	12	I45-56
National timber trade (e.g., GD Holz, UK TTF), timber retail (e.g., IKEA, OTTO, OBI) and forest industry companies and their European associations (e.g., ATIBT, ETTF, CeiBoi, CEPI)	25	I57-81
Research and think-tanks (e.g., EFI-FLEGT/REDD Facility, Chatham House UK) and certification bodies (e.g., FSC DE, FSC UK and FSC International)	5	I82-86
**Total number:**	86	

Table S4: List of policy documents subject to content analysis, EUTR/FLEGT, 1998-2020

Document Type	Year of Publication	Author, Institution	Title (Abbreviation used in the main text)
Policy paper	1998	G8 governments	Text: Joint G8 Statement on Forest Management. Action plan adopted to protect world's forests. 11.05.1998 (D1)<br>http://www.usembassy-israel.org.il/publish/press/trade/archive/1998/may/et3512.htm
Policy paper	2002	UK Forest Partnership for Action	A new partnership within the UK of Government, business and environmental groups to promote sustainable development in the forest sector, both at home and internationally. http://www.forestry.gov.uk/pdf/ukforestpartnership.pdf (D2)
Policy paper	2006	SKANSKA Group and a Group of Forest Industries and Retailers	FLEGT: INDUSTRY STATEMENT Common European rules for fair competition and sustainable markets, 16.03.2006 (D3)<br>http://group.skanska.com/globalassets/sustainability/environmental-responsibility/materials/sustainability-of-materials/flegtindustry.pdf
Policy paper	2006	Greenpeace International and 164 other national and international environmental NGOs and 24 individuals from Europe, Russia and Asia (e.g., Indonesia, Malaysia)	Controlling Timber Imports into the EU, Joint NGO Statement, 19.09.2006 (D4).<br>http://www.greenpeace.org/international/Global/international/planet-2/report/2006/9/controlling-timber-imports-int-2.pdf
Policy paper	2007	WWF UK, and ca. 90 forest industry organisations	FLEGT: Industry Statement. WWF UK, 21.05.2007 (D5) http://assets.panda.org/downloads/industrystatement6.pdf
Policy paper	2007	Greenpeace	Partners in crime: How Dutch Timber Traders Break their Promises, Trade Illegal Timber and Fuel Destruction of the Paradise Forests, Greenpeace Netherlands, April 2007. (D6)

Legislative proposal	2008	European Commission	COM(2008) 644 final, 2008/0198 (COD), C6-0373/08, Commission of the European Communities: Proposal for a Regulation of the European Parliament and of the Council laying down the obligations of operators who place timber and timber products on the market, Brussels, 17.10.2008 (D7)
Legislative proposal	2008	Government of Netherlands	13497/08, Note from the Netherlands delegation to Council, Page 3, Brussels, 26.9.2008 (D8)
Press release	2008	Council of European Union	13522/08 (C/08/274), Press Release 2892nd meeting of the Council Agriculture and fisheries, Page 15, Brussels, 29-30.9.2008 (D9)
Press release	2008	FEBO	Questions raised about EC illegal timber plans. European Timber Trade Association (FEBO). Timber Trades Journal Online. 28.11.2008. (D10)<br>http://www.ttjonline.com/news/questions-raised-about-ec-illegal-timber-plans/ (accessed 15.05.2013)
Policy paper	2008	EUSTAFOR	EUSTAFOR position paper timber products 16 July 2008. (D11) http://www.eustafor.eu/failid/File/Position%20Papers/EUSTAFOR%20position%20paper%20illegal%20logging_16_07_2008.pdf
Policy paper	2008	Greenpeace	Briefing on Commission Proposal against Illegally Harvested Timber, Greenpeace International, July 2008. (D12)<br>www.greenpeace.org
Legislative proposal	2009	European Parliament	A6-0115/2009, Report on the proposal for a regulation of the European Parliament and of the Council laying down the obligations of operators who place timber and timber products on the market (COM(2008)0644 – C6-0373/2008 – 2008/0198(COD). European Parliament, Committee on the Environment, Public Health and Food Safety, 02.03.2009 (D13)
Policy paper	2009	Friends of the Earth	Environment groups: MEPs must improve EU timber law. Friends of the Earth, 16.02.2009. (D14)

			http://www.foeeurope.org/press/2009/Feb16_MEPs_must_improve_EU_timber_law.html
Press release	2009	Greenpeace	Greenpeace statement on the European Parliament Environment Committee vote on the EU timber law. 7.02.2009. (D15) http://www.greenpeace.org/eu-unit/en/News/2009-and-earlier/statement-vote-timber-law090217/ (accessed 29.05.2013)
Press release	2009	Council of European Union	5471/09 (Press 13), 2918th meeting of the Council Agriculture and Fisheries, Brussels, 19.02.2009 (D16)
Legislative proposal	2009	European Parliament	8881/09, Note from General Secretariat to Permanent Representatives Committee/Council, Outcome of the European Parliament's first reading, Brussels, 27.04.2009 (D17)
Legislative proposal	2009	UK Government	9027/09, Note from UK delegation to Council Working Party on Forestry, Brussels, 27.04.2009 (D18)
Legislative proposal	2009	Council of European Union	10076/09, Note from General Secretariat of the Council to Working Party on Forestry, Brussels, 19.05.2009 (D19)
Legislative proposal	2009	Council of European Union	16529/09, Report from Working Party of Counsellors/Attachés to Permanent Representatives Committee (Part I), Brussels, 27.11.2009 (D20)
Legislative proposal	2009	UK and Denmark	17531/09, NOTE from UK and DK delegation to Council, Brussels, 14 December 2009 (D21)
Legislative proposal	2009	Governments of UK, Denmark, Belgium, Spain	17632/09, Note from UK, DK, BE, ES delegations to Council, Brussels, 16.12.2009 (D22)
Press release	2009	Friends of the Earth	European Parliament votes for strong law to stop illegal timber trade. Friends of the Earth. 22.04.2009 (D23)<br>https://foeeurope.org/press/2009/Apr22_EP_votes_for_strong_law_to_stop_illegal_timber_trade.html

Press release	2009	Greenpeace	European governments reject stronger legislation on illegal logging. Greenpeace. 15.12.2009 (D24).<br><br>http://www.greenpeace.org/eu-unit/en/News/2009-and-earlier/EU-illegal-timber-logging-legislation-15-12-09
Press release	2009	EFPI	EFPI introduces Code of Conduct on Sustainability. European Federation of Parquet Importer. 06.12.2009. (D25)<br><br>http://www.illegal-logging.info/item_single.php?it_id=3969&it=news
Legislative proposal	2010	European Parliament	A7-0149/2010, Recommendation for Second Reading, Page 57, European Parliament, 07.05.2010 (D26)
Press release	2010	UK Timber Trade Federation	UK timber industry unites to urge the European Parliament and Council to agree a workable but strong Due Diligence regulation. Timber Trade Federation. 29.04.2010. (D27)<br><br>http://www.confor.org.uk/NewsAndEvents/News.aspx?pid=24&id=65
Press release	2010	NEPCon	Ban on illegal timber in EU - pros and cons. NEPCon. 17.02.2010. (D28) http://www.nepcon.net/3193/English/HOME/News_2010/February/Ban_on_illegal_timber_in_EU_-_pros_and_cons/
Press release	2010	Greenpeace	EU bans illegal timber - Strong legislation follows ten year Greenpeace Campaign. Greenpeace. 07.07.2010. (D29) http://www.greenpeace.org/eu-unit/en/News/2010/eu-bans-illegal-timber/ (accessed 16.05.2013)
Legislation in force	2010	European Parliament and Council of the European Union	Regulation (EU) No 995/2010 of the European Parliament and of the Council of 20 October 2010 laying down the obligations of operators who place timber and timber products on the market. 12.11.2010. Official Journal of the European Union L 295, pp.23-34 (http://eur-lex.europa.eu/legal-content/EN/TXT/?uri=CELEX:32010R0995)

			(D30)
Press release	2010	Kingfisher, IKEA, B&Q, CEPI	Kingfisher forms Timber Retail Coalition, 6.04.2010. (D31) http://www.kingfisher.com/index.asp?pageid=55&newsid=854
Evaluation report	2016	European Commission	REPORT FROM THE COMMISSION TO THE EUROPEAN PARLIAMENT AND THE COUNCIL Regulation EU/995/2010 of the European Parliament and of the Council of 20 October 2010 laying down the obligations of operators who place timber and timber products on the market (the EU Timber Regulation). COM/2016/074 final (D32)
Policy and legislative document	2019	European Commission	COMMUNICATION FROM THE COMMISSION TO THE EUROPEAN PARLIAMENT, THE COUNCIL, THE EUROPEAN ECONOMIC AND SOCIAL COMMITTEE AND THE COMMITTEE OF THE REGIONS. Stepping up EU Action to Protect and Restore the World's Forests. COM/2019/352 final (D33)
Public consultation and written survey responses	2020	European Commission (39 written feedbacks from environmental NGOs and forest sector stakeholders received between 31 January 2020 and 28 February 2020)	Public consultation "Illegal logging – evaluation of EU rules" / Fitness Check of the EU Timber Regulation (D34)<br>https://ec.europa.eu/info/law/better-regulation/have-your-say/initiatives/11630-Illegal-logging-evaluation-of-EU-rules-fitness-check-_en
Legal proposal	2020	European Parliament	Deforestation – European Parliament resolution of 20 October 2020 with recommendations to the Commission on an EU legal framework to halt and reverse EU driven global deforestation (2020/2006/INL) (D35)

Table S5: List of key informant interviews, EU climate policy in the LULUCF sector, 2018-2020

**Interviewee's affiliation**	**Number**	**Abbreviations used in the text**
European Commission, DG Environment<br><br>European Commission, DG Climate Action<br><br>European Commission, DG Agriculture and Rural Development	1<br><br>2<br><br>1	I1<br><br>I2-3<br><br>I4
European Parliament, COM-ENVI (1x conservative, 1x green party)	2	I5-6
State environmental and forestry authorities of EU Member States (Austria, France, Finland, Germany, Ireland, Hungary, Poland, Spain, Sweden)	12	I7-18
National non-state forest owners, state forestry enterprises and farmers organisations (FNCOFOR, Fransylva, Bauernverband) and their European associations (EUSTAFOR, CEPF, ELO, Copa-Cogega)	7	I19-25
European associations of forest industries (Cei-Bois, CEPI)	2	I26-27
National (FNE, NABU) and European (BirdLife, FERN, CAN, WWF) environmental and climate NGOs	5	I28-32
National and European researchers and think-tanks (JRC, EEA, LUKE, ECOFOR, CITEPA, CEP)	6	I33-38
**Total**	38	

Table S6: List of policy documents subject to content analysis, EU climate policy in the LULUCF sector, 2018-2020

Document Type	Year of Publication	Author, Institution	Title (Abbreviation used in the tex)
Impact assessment/ Legislative proposal	2014	European Commission	Commission staff working document impact assessment. Communication from the Commission to the European Parliament, the Council, the European Economic and Social Committee and the Committee of the Regions A policy framework for climate and energy in the period from 2020 up to 2030. {COM(2014) 15 final} {SWD(2014) 16 final}. European Commission (2013). (D1)<br>http://eur-lex.europa.eu/legal-content/EN/TXT/PDF/?uri=CELEX:52014SC0015&from=EN.
Legislative proposal	2014	European Parliament	A 2030 framework for climate and energy policies. European Parliament resolution of 5 February 2014 on a 2030 framework for climate and energy policies (2013/2135(INI)) (D2)
Legislative proposal	2014	European Council	Conclusions on 2030 Climate and Energy Policy Framework. European Council (2014): European Council (23 and 24 October 2014 - Conclusions). In European Council (October). (D3)<br>DOI: 10.1007/s13398-014-0173-7.2.
Fact sheet	2016	European Commission	European Commission - Fact sheet. Proposal to integrate the land use sector into the EU 2030 Climate and Energy Framework. (D4)
Policy paper	2016	Bird Life International	“Toward sustainable mitigation in agriculture and land use”. (D5)
Position paper	2016	EUSTAFOR	EUSTAFOR Position Paper on the Role of Forests and Forest Products in the Post-2020 EU Climate Change Policy Framework. How can European state forests contribute to post-2020 EU climate policy targets? (D6)
Policy evaluation	2016	JRC (Joint Research Center of the EU Commission)	The EU greenhouse gas inventory for the LULUCF sector: I. Overview and comparative analysis of methods used by EU member states. (D7)<br>DOI: 10.1080/17583004.2016.1151504.
Policy paper	2016	CAN (Climate Action	NGO Position on the Post-2020 LULUCF Regulation. (D8) http://www.caneurope.org/docman/land-

		Network Europe)	based-emissions/3023-can-europe-lulucf-position-dec-2016/file
Legislative proposal	2016	European Commission	Regulation of the European Parliament and of the council. on the inclusion of greenhouse gas emissions and removals from land use, land use change and forestry into the 2030 climate and energy framework and amending Regulation No 525/2013 of the European Parliament and the Council on a mechanism for monitoring and reporting greenhouse gas emissions and other information relevant to climate change. {SWD(2016) 246 final} {SWD(2016) 249 final}. European Commission. (D9)
Legislative proposal	2016	European Commission	REGULATION OF THE EUROPEAN PARLIAMENT AND OF THE COUNCIL. on the inclusion of greenhouse gas emissions and removals from land use, land use change and forestry into the 2030 climate and energy framework and amending Regulation No 525/2013 of the European Parliament and the Council on a mechanism for monitoring and reporting greenhouse gas emissions and other information relevant to climate change. (D10)
Position papers	2017	CEPF	CEPF Position Paper on the proposal for a LULUCF Regulation. (COM(2016) 479). Available online at http://www.cepf-eu.org/news/eu-agrees-lulucf-regulation, updated on 6/21/2018, checked on 6/21/2018. (D11)<br><br>LULUCF: Forestry, paper and agrisectors team up in favor of a dynamic forest reference level. http://www.cepf-eu.org/news/lulucf-forestry-paper-and-agri-sectors-team-favor-dynamic-forest-reference-level, checked on 6/21/2018. (D12)
Legislative proposal	2017	Council of the European Union	Proposal for a Regulation of the European Parliament and of the Council on the inclusion of greenhouse gas emissions and removals from land use, land use change and forestry into the 2030 climate and energy framework and amending Regulation No 525/2013 of the European Parliament and the Council on a mechanism for monitoring and reporting greenhouse gas emissions (First reading). (D13)
Policy paper	2017	EEB; WWF; CAN; TE; Carbon	Lettre ouverte au ministre français de la transition écologique et solidaire sur la position de négociation de la france à l’égard du règlement sur la répartition de l’effort et du règlement sur l’utilisation des

		Market Watch; Fern	terres, le changement d'affectation des terres et la foresterie. (D14)
Policy paper	2017	A group of 190 scientists	Scientific basis of EU climate policy on forests. (D15) https://drive.google.com/file/d/0B9HP_Rf4_eHtQUpyLVIzZE8zQWc/view
Policy paper	2018	CAN (Climate Action Network Europe)	LULUCF: rankings reveal member states' forest policy positions. (D16)<br>http://www.caneurope.org/component/content/article?id=1386:lulucf-rankings-reveal-member-states-forest-policy-position, checked on 6/18/2018.
Public consultation	2018	European Commission	Consultation on addressing greenhouse gas emissions from agriculture and LULUCF in the context of the 2030 EU climate and energy framework - Climate Action - European Commission. https://ec.europa.eu/clima/consultations/articles/0026_en, updated on 8/20/2018, checked on 8/21/2018. (D17)
Fact sheets	2018	European Commission	Land use and forestry regulation for 2021-2030 - Action pour le climat - European Commission. European Commission. Available online at https://ec.europa.eu/clima/lulucf_fr#tab-0-0, updated on 6/18/2018, checked on 6/18/2018. (D18)
Regulation	2018	European Parliament and Council of the European Union	Regulation (EU) 2018/841 of the European Parliament and of the Council of 30 May 2018 on the inclusion of greenhouse gas emissions and removals from land use, land use change and forestry in the 2030 climate and energy framework, and amending Regulation (EU) No 525/2013 and Decision No 529/2013/EU (Text with EEA relevance) (D19)
Legislative proposal / Policy Strategy	2019	European Commission	COMMUNICATION FROM THE COMMISSION TO THE EUROPEAN PARLIAMENT, THE EUROPEAN COUNCIL, THE COUNCIL, THE EUROPEAN ECONOMIC AND SOCIAL COMMITTEE AND THE COMMITTEE OF THE REGIONS. The European Green Deal. COM/2019/640 final (D20)
Legislative Proposal	2020	European Parliament and Council of the European Union	Proposal for a REGULATION OF THE EUROPEAN PARLIAMENT AND OF THE COUNCIL establishing the framework for achieving climate neutrality and amending Regulation (EU) 2018/1999 (European Climate Law). COM/2020/80 final (D21)

Legislative proposal / Policy Strategy	2020	European Commission	COMMUNICATION FROM THE COMMISSION TO THE EUROPEAN PARLIAMENT, THE COUNCIL, THE EUROPEAN ECONOMIC AND SOCIAL COMMITTEE AND THE COMMITTEE OF THE REGIONS. Stepping up Europe’s 2030 climate ambition Investing in a climate-neutral future for the benefit of our people. COM/2020/562 final (D22)
